# ISOBlue HD: An Open-Source Platform for Collecting Context-Rich Agricultural Machinery Datasets

**DOI:** 10.3390/s20205768

**Published:** 2020-10-12

**Authors:** Yang Wang, He Liu, James Krogmeier, Amy Reibman, Dennis Buckmaster

**Affiliations:** 1 Electrical and Computer Engineering, Purdue University, West Lafayette, IN 47907, USA; liu1433@purdue.edu (H.L.); jvk@purdue.edu (J.K.); reibman@purdue.edu (A.R.); 2 Agricultural and Biological Engineering, Purdue University, West Lafayette, IN 47907, USA; dbuckmas@purdue.edu

**Keywords:** precision agriculture, machinery, open-source, ISOBUS, controller area network, data collection, context

## Abstract

This paper introduces an open-source platform called *ISOBlue HD* for acquisition of context-rich data from agricultural machinery. We call these datasets context-rich, because they enable the identification of machine status and farming logistics by properly labeling, fusing, and processing the data. The system includes a single board computer, a cellular modem, local storage, and power-over-ethernet switch to sensors. The system allows remote diagnostics and access, automatic startup/shut down with vehicle operations, and uses Apache Kafka to enable robust data exchange. *ISOBlue HD* was deployed in a combine harvester during a 2019 wheat harvest for simultaneously capturing 69 million CAN frames, 230,000 GPS points, and 437 GB of video data, focusing on header status and operator actions over 84 h of harvest time. Analyses of the collected data demonstrate that contextual knowledge can be inferred on harvest logistics (paths, speeds, header status, material transfer) and sensor data semantics.

## 1. Introduction

At its core, modern agriculture is a business of logistics where farmers find their competitive advantage relative to other farmers in the timeliness and efficiency with which they make informed decisions in preparing the soil, applying crop nutrients, planting the seed, applying crop protection and water, harvesting, and marketing the crop. The choices they make in inputs such as fertilizer, seed genetics, and chemicals, while important, are secondary to timeliness and the weather. Therefore, the first adopted and most widely used tools of precision agriculture were those which improved timeliness and efficiency, such as advanced satellite positioning technologies, auto-steer, larger implements, faster working speeds, and business and market information enabled by the internet. Viewed as a critical part of agriculture-as-logistics, the performance of distributed agricultural systems comprised of multiple interacting machines and human operators is of critical importance. See [1,2,3,4,5,6,7].

Much of the relevant agricultural machine data are transported over the wired ISOBUS network [8] running the controller area network (CAN) [9] protocol. An agricultural machine typically has two CAN busses: the tractor bus and the implement bus. Electronic control units (ECUs) use this network to communicate with each other and to receive and transmit data from many sensors. Data of interest include machine status information like engine speed, or operation-dependent information, like instantaneous crop flow rate, crop moisture readings, application rate, etc.

The promise of machine learning (ML) and artificial intelligence (AI) [10] in the continued automation of agricultural machinery [11,12,13,14,15] cannot be realized without new high bandwidth sensors such as video and lidar, high performance on-machine networking, on-machine edge computing, and reliable Cloud connection. In addition, and perhaps most daunting is the need for huge sets of synchronously logged machine, positioning, video, and lidar data, which have been cleaned, clipped, and labeled for training. Traditional manual processes [16] for data retrieval and wrangling are woefully inadequate. Before autonomous machines can be perfected, there needs to be a development of the data networking and computational systems that allow automation of the data cleansing, segmentation, and context labeling pipeline. In other words, the design and training of autonomous machines will require autonomy in the training data pipeline. This task will also rely upon advances in ML and edge computing [17].

In this paper, the term “context” is understood to mean labels of datasets, which are relevant for training of machine learning algorithms or for other types of statistical analysis. The formalization of the “context” notion has been in the works for quite some time, since the advent of context-aware computing [18], but for simplicity, this paper will stick to “context” related to wheat harvest operations involving the interaction of combine harvesters, tractors pulling grain carts, trucks hauling grain from fields to receiving facilities, and the people operating them. Taking the combine harvester as an example, context refers to what the machine-operator is doing at a particular moment. For example: (1) a combine may be moving back and forth in a field harvesting wheat, (2) a combine may be stopped and unloading on a grain cart or truck, (3) a combine may be unloading on a grain cart while it is simultaneously harvesting grain (called “unloading on the go”), (4) a combine may be cornering or maneuvering outside of the unharvested field area, (5) a combine may be traveling on a road, (6) a combine may be maneuvering around an in-field obstacle, (7) a combine may be idling or otherwise not operational.

These contexts, which are easily understood by human operators, directly affect operators’ in-field decisions. One of the key tactical decisions, for instance, is the choice of path. Proprietary work by OEMs and research studies have made great progress towards route optimizations, as seen in [19,20,21,22]. While often such optimization algorithms may suffice, this is not always the case. The use of real-time and recent-past data can facilitate performance by reducing wasted time, travel distance, and fuel usage. To truly optimize, it will require full integration of CAN (yield, speed, engine load, etc.), Global Navigation Satellite System (GNSS) tracks, video, and additional information like topography and audio. In the move toward autonomy, such comprehensive datasets are required, as they contribute to robustness in AI developments but, more immediately, enable improved decision-making assistance (e.g., route suggestion, anomaly detection) for operators. Hence, while the combine is operating, sensor data are typically being captured, for which the meta-data about context is needed to explain and properly label the datasets. In this paper, a dataset that contains sufficient information for algorithms to label, fuse, mine, and infer a full suite of contexts related to machine and surroundings, is referred to as a context-rich dataset. The focus of this paper is to collect such data; it leads to the creation of an open-source platform called *ISOBlue HD* for capturing CAN, GNSS, and video data from a combine harvester during wheat harvest operations. Additionally, this paper intends to demonstrate exemplary data processing steps in analyzing a context-rich dataset from a realistic deployment.

Related works in terms of machinery data collection are reviewed in Section 2. Details on hardware and software implementations of *ISOBlue HD* are provided in Section 3 and Section 4. Moreover, a 2019 wheat harvest data collection experiment using *ISOBlue HD* is described in Section 5, along with proof-of-concept results for context-rich visualizations and analyses. Finally, future research directions are discussed in Section 6.

## 2. Related Works

**Figure 1:**
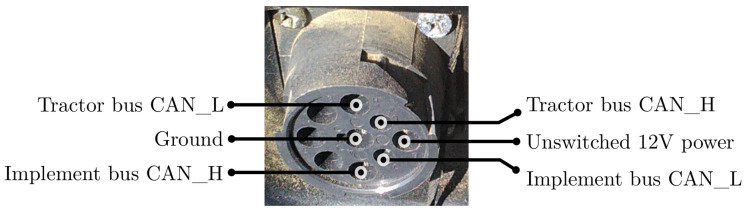
An ISOBUS diagnostic port is an access point for hardware tools to collect CAN data and from modern agricultural machinery.

For collecting data from modern agricultural machinery, a widely accepted method is to use an ISOBUS diagnostic port [23], as shown in Figure 1. The diagnostic port provides both power and CAN bus data. This port is commonly utilized in machine maintenance, for attaching service and diagnostic tools for monitoring machine and implement statuses [24].

Commercial loggers, backed by their powerful software suites, are favorable choices among researchers for capturing CAN data via the ISOBUS diagnostic port for machine-centric analysis. For instance, a commercial logger was configured in [25] to log GNSS data, to determine field boundaries for plowing operations. Moreover, three commercial loggers were evaluated in [26] for their feasibilities in real-time engine speed and load analysis. Nevertheless, with the increasing popularity of affordable wireless and sensor technologies, researchers find it more versatile to create custom hardware platforms using readily available hardware components for machinery applications [27]. A selection of such recent platforms is listed in Table 1.

Platforms like CyCAN [28], deployed in past experiments like [35,36], can collect CAN data only. Other platforms, namely ISOBlue 2.0 [31] and Cropinfra [34], are able to capture GNSS data in addition to CAN data. However, none of these platforms collect rich-enough data to infer operational contexts. On the other hand, the FieldSafe platform [32] presents a method that uses a wide variety of sensors and ranging devices to capture geospatial, video, and ranging data during tractor mowing operations. Although the collected dataset might reflect contexts related to in-field obstacle avoidance, it does not contain CAN data.

No platform has been created to collect a dataset that contains both detailed machine information (CAN and GNSS) with minable content for inferring contexts. *ISOBlue HD* is designed to fill this gap with a set of cameras in addition to CAN modules and GNSS receivers; the reason for choosing cameras is that there is no similar sensor that offers both cost-effectiveness and direct visual perception of what has happened. The making of *ISOBlue HD* involves both hardware selection and software development. For hardware, the device is powered directly via an ISOBUS diagnostic port. Moreover, it is installed with a cellular modem for Internet connection. Furthermore, the device is enclosed in a durable enclosure to withstand dust and vibration. Software development is open-source and is built on software stacks from previous projects (*ISOBlue* [29] and *ISOBlue 2.0* [31]). Logger programs are developed to log CAN, GNSS, and video data simultaneously once the machine starts. Power manager programs also help the device wake up and suspend according to the machine on/off state. Moreover, diagnostic data need to be logged and synchronized periodically to a remote Cloud instance for monitoring purposes. Last, but not least, a debugging interface is also developed to provide remote access to the device during deployment.

## 3. Hardware Components

**Figure 2:**
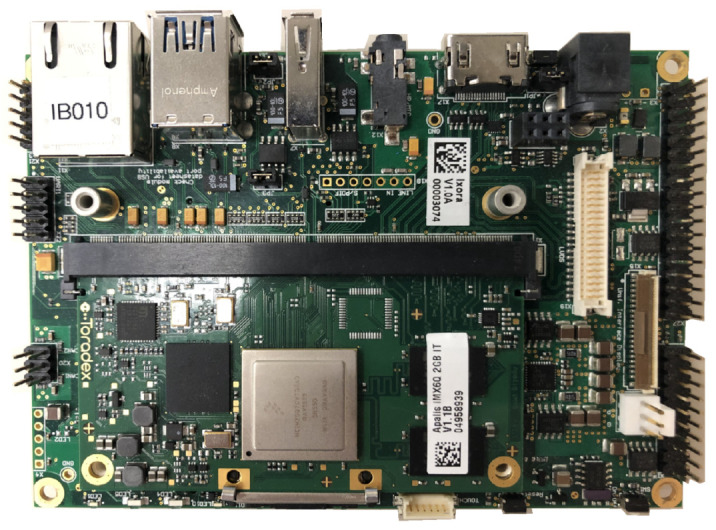
A single board computer was selected as the core computing hardware.

A single board computer shown in Figure 2 was chosen as the core computing hardware. Its specifications [37] are highlighted in Table 2. The board was chosen for a few reasons. First, the on-board quad-core processor, coupled with 2 GB of RAM, provided plentiful computing power for logging tasks. Secondly, the on-board power regulator supported a wide input power range from 7 to 27 V, which is compatible with the diagnostic port’s power without extra power conversion. Thirdly, the board came with a number of peripherals and connectors for interfacing with additional hardware components. This not only satisfies the peripheral needs for this experiment, but also opens up possibilities for bidirectional communication with new sensors or even in-cab information systems like yield monitors in the long run.

**Figure 3:**
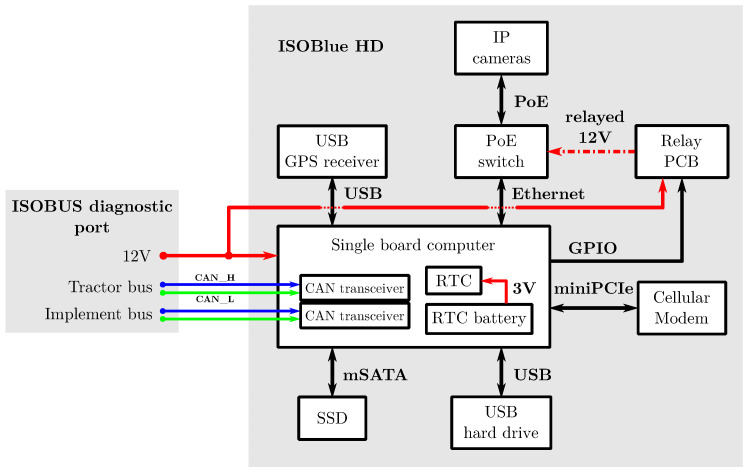
*ISOBlue HD* connects to a machine’s ISOBUS diagnostic port for both power and CAN bus signals. The single board computer interfaces with various components for data collection, data storage, sensor control, and cellular connectivity.

A systematic overview on how the single board computer interfaces with other hardware components is given in Figure 3. *ISOBlue HD* connects directly to an ISOBUS diagnostic port for both power and CAN bus connections. Sensors, cellular module, and hard drives are either built-in or connected to the single board computer for sensing, storing, and streaming data. In regard to power distribution, most components are powered directly through connected peripherals (mSATA, USB, and miniPCIe) from the single board computer. However, there are two exceptions: (1) the on-board real time clock (RTC) is powered by a standalone 3 V battery and (2) the internet protocol (IP) cameras rely on a power-over-ethernet (PoE) network switch and a custom relay PCB to receive GPIO-controlled power.

### 3.1. Data Sources

**Figure 4:**
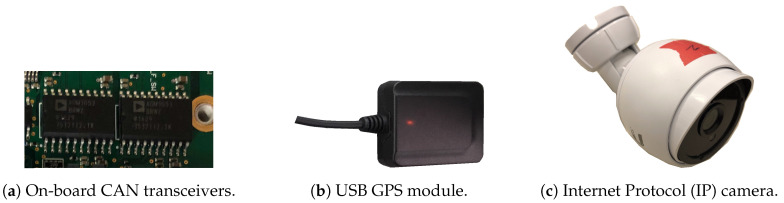
Three types of electronic components are utilized for collecting CAN, GNSS, and video data. Detailed specifications of these components are given in Table 3.

Three types of electronic components illustrated in Figure 4 were utilized as data sources for CAN, GNSS, and video data. Their specifications are provided in Table 3. For sensing CAN signals, the single board computer came with two identical CAN transceivers. Each transceiver was configured to have a baud rate of 250 kbps for converting CAN bus signals into bitstreams. The bitstreams were further processed by custom programs to construct CAN frames. Each CAN frame was roughly 12 bytes (with the extended CAN frame format). Hence, with a nominal acquisition rate of 700 frames per second, around 8.4 kB of CAN data were written to the disk each second.

For receiving GNSS data, a USB GPS module was utilized. Upon the reception of stable satellite signals, the GPS module reported new GPS fixes at 1 Hz. The fixes included both geospatial and accuracy data, which adds up to around 26 bytes. In addition, the GPS module sent pulse per second (PPS) signals over USB. These signals were utilized for keeping system time accurate.

Video data were collected via three IP cameras. The tri-camera setup aimed to capture video data from various viewing angles. The cameras supported the real time streaming protocol (RTSP) [41] and they were configured as dynamic host configuration protocol (DHCP) clients to make video streams accessible via IP addresses. Each video stream had a resolution of 1920 × 1080 at 30 fps, with an encoding rate of 6000 kbps. Moreover, the cameras and the single board computer established a local area network (LAN) through a veracity power-over-ethernet (PoE) network switch [42]; it supplied IEEE 802.3af [43]-compliant electric power to the cameras and bridged data connection for the LAN via ethernet cablings. By using wired connections, higher bandwidths and therefore better video quality can be achieved.

### 3.2. Additional Components

**Figure 5:**
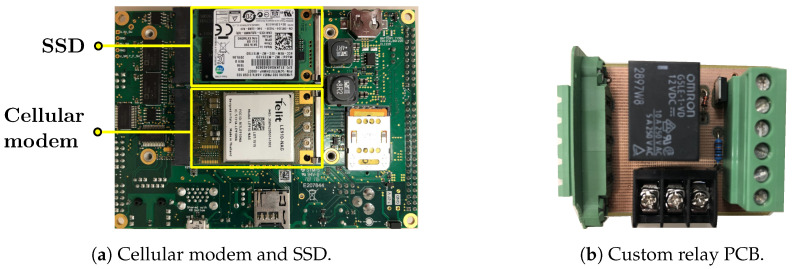
Additional components interface with the single board computer for Internet access, data storage, and power control features.

A Telit LE910 [44] cellular modem (Figure 5a) was installed with two patch antennas for 4G/LTE network access. In terms of data storage, two storage devices were included: a 512 GB mSATA solid state drive (SSD) (Figure 5a) and a 4 TB USB hard drive. The main purpose of the SSD was to store CAN and GNSS data. On the other hand, the USB hard drive was dedicated to store all video data, as they would take more disk space. Moreover, a custom-printed circuit board (PCB) (Figure 5b) was created for controlling power to the PoE network switch and the cameras. The PCB was controlled by a GPIO pin from the single board computer. The toggling of the pin was based on the power state of the single board computer. Each time the board went to suspend, the GPIO pin was set to low, which signaled the relay PCB to cut the 12 V off to the network switch. Conversely, the relay PCB resumed the 12 V to the network switch, when the board was on and the pin was set to high.

### 3.3. Enclosure

**Figure 6:**
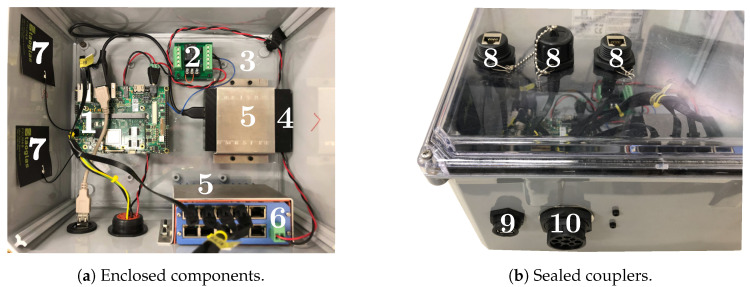
Hardware components are housed in a sealed enclosure to withstand dust and vibrations, as shown in (**a**). Couplers shown in (**b**) provide sealed connections between internal and external components. The annotated components are: (1) single board computer (2) relay PCB (3) plexiglass base (4) USB hard drive (5) anti-vibration straps (6) PoE switch (7) cellular antennas (8) Ethernet couplers (9) USB coupler and (10) ISOBUS coupler.

All hardware components except the USB GPS module and the cameras were securely housed in dust- and water-resistant enclosure, as shown in Figure 6a. A custom-made plexiglass plate was placed at the bottom of the enclosure as the base for mounting hardware components. For CAN, USB, and ethernet connections, three types of couplers shown in Figure 6b provided sealed connections for interconnecting internal and external components. Although both the enclosure and the couplers are rated ingress protection (IP) [45] 67, the platform as a whole does not meet strictly to any protection standard; it is a viable solution for this experiment, as the primary focus for having an enclosure is to minimize the chance of hardware damage, which could lead to data corruption during deployment. A complete bill of materials (BOM) with unit pricings for building an *ISOBlue HD* is given in Table A1. Readers are encouraged to reproduce and customize the hardware platform for their own applications.

## 4. Software Development

**Figure 7:**
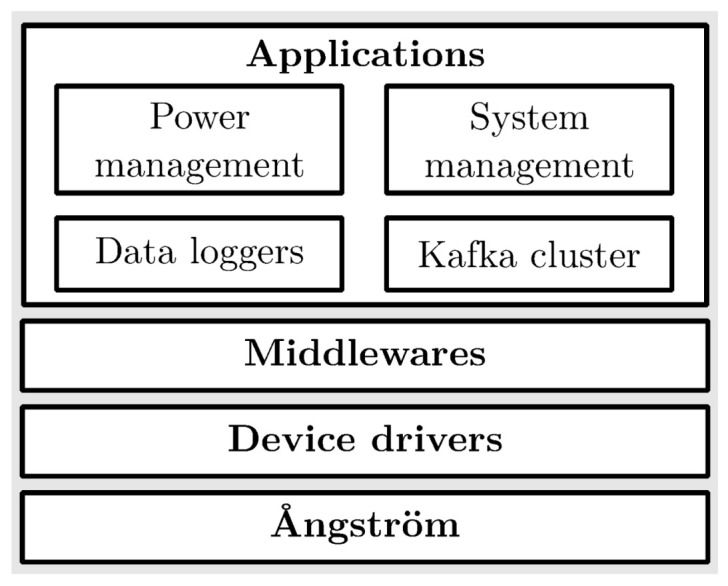
The custom Board Support Package (BSP) contains four areas of applications running on an operating system called Ångström: system management, power management, data cluster, and data logging.

The software implementations focus on the customizations of an open-source board support package (BSP). As overviewed in Figure 7, the BSP contains Ångström [46]—an lightweight operating system, applications that encapsulate system management programs, power management programs, a Kafka cluster, and data loggers with additional device drivers and middlewares. Custom applications were written in a generic fashion that could be easily ported to another platform if needed. All source code was developed under the Apache 2.0 license and is available on GitHub [47].

### 4.1. System Management

Some existing open-source applications listed in Table 4 were utilized for managing system hardware and application executions. For managing hardware, *udev* [48] employed custom rules to automatically monitor hardware and triggered actions upon hardware changes. Three custom *udev* rules were written to: (1) bring up two CAN interfaces with the correct baud rates, (2) enable wake-on-CAN feature, and (3) establish cellular connections with an access point name (APN). Moreover, a GPS service daemon called *gpsd* [49] was installed for interfacing with the GPS module. This daemon periodically fetched data from the GPS module and made them available on a TCP port. For overseeing and automating program executions, *systemd* [50] was utilized. Applications used dedicated *systemd* service files to specify execution orders, dependencies, start/restart policies.

On the network side, *openssh* [51] was utilized to port-forward a local port to a remote desktop. This enabled remote access to the platform for debugging purposes. Besides, the single board computer was configured as a DHCP server to communicate with the cameras via the PoE switch using *dnsmasq* [52]. This tool utilized a configuration file that specifies: (1) a server IP address, (2) a list of MAC addresses of the cameras, and (3) an IP address range. Whenever the cameras were on, they automatically sent DHCP requests for becoming clients. Once *dnsmasq* received the requests, it would automatically assign IP addresses using the specified range. As soon as this process finished, video loggers could access camera streams via their IP addresses.

For system time-keeping, *chrony* [53] was configured to correct system time according to reliable time sources. The application was supplied with two time sources: (1) a list of network time protocol (NTP) servers and (2) the PPS source from the USB GPS module. These two sources provided a fail-safe way to synchronize time, regardless of Internet availability.

### 4.2. Power Management

**Figure 8:**
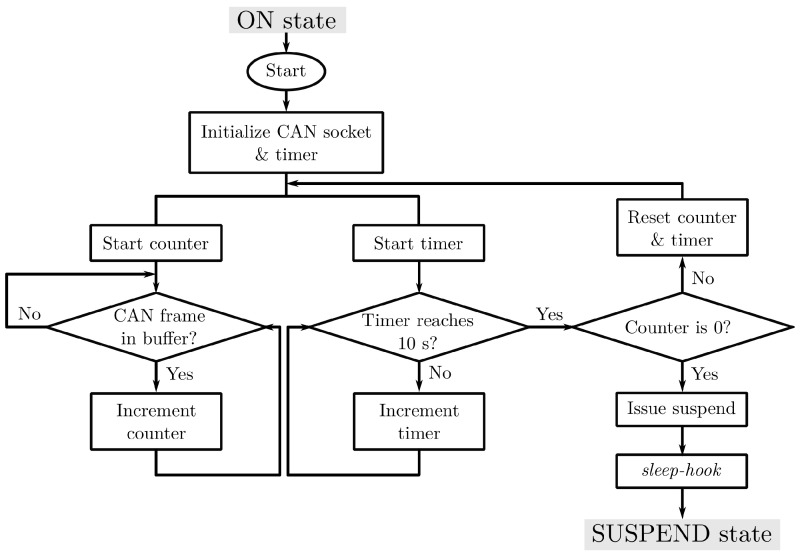
A custom application called *can-watchdog* manages system power by checking the number of CAN frames received within a resettable time period. Whenever no CAN frame is received within the timer period, *can-watchdog* issues a command that suspends the board and triggers *sleep-hook* to toggle the GPIO pin to low for cutting the power to the PoE switch and the cameras.

Two applications, *can-watchdog* and *sleep-hook*, were written to control the power of the hardware according to the machine power state. The workflow of the applications is visualized in Figure 8. Specifically, *can-watchdog* employs a combination of a Linux timer and SocketCAN [54]—a kernel driver that implements CAN protocol for Linux and provides CAN interfaces as network sockets. Once the application starts, it initializes two CAN sockets and a 10-s timer. Then, it enters into a loop that continuously listens for incoming CAN frames and resets the timer if there are new CAN frames. If the application sees no incoming CAN frame for 10 s, it would issue a suspend command, which also triggers *sleep-hook* to set the GPIO pin to low. This scheme both suspends the single board computer and signals the relay to shut off power to the PoE switch and cameras.

After the suspend, the single board computer saves all system states in its memory; leaving one working CPU core and the CAN transceivers on. Whenever the machine is turned back on, CAN activity would resume. The working core is sent an interrupt by the CAN transceiver, waking up the board. This triggers *sleep-hook* to set the state of the GPIO pin to high, which turns back on the PoE switch and the cameras.

### 4.3. Kafka Cluster

**Figure 9:**
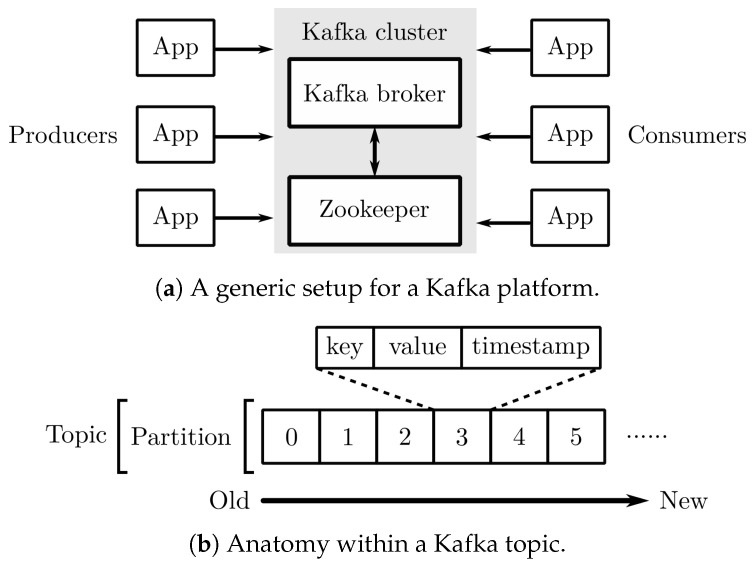
The setup in (**a**) shows a basic Kafka setup that consists of a Kafka cluster and clients. The Kafka cluster is comprised of a *Kafka broker* and a *Zookeeper*. Kafka clients either push (producers) or pull (consumers) data to or from a Kafka broker. The data are stored as immutable records within a Kafka topic, as shown in (**b**).

Apache Kafka [55] was chosen to log CAN, GNSS, and diagnostic data for its robust data exchange between applications and systems [56,57]. The basic components of Kafka contain a Kafka cluster and Kafka clients (producers and consumers), as shown in Figure 9a. A Kafka cluster consists of two components: a *Kafka broker* and a *Zookeeper*. They work jointly to coordinate with clients for data storing and distribution. Data, referred to as records in Kafka’s convention, are stored in different topics. The topics for storing CAN, GPS, and diagnostic data are listed in Figure 9b. Moreover, all records in Kafka topics are saved in an immutable and append-only sequence. The immutability guarantees the order of the data on the disk. As the data of interest are all time-series, this feature is particularly helpful as it prevents potential data shuffling.

The Kafka software was downloaded from Apache Kafka’s archive [58]. An instance of *Kafka broker* and *Zookeeper* run to form a Kafka cluster. The cluster manages four Kafka topics in Table 5. Data loggers described later in Section 4.4 connect to the cluster as Kafka producers to publish sensor data to these topics. Moreover, a Kafka software called *MirrorMaker* synchronizes data stored in the *remote* topic to a remote Kafka cluster via a protected SSH tunnel. The synchronized data provide diagnostic and geospatial information of the platform for visualization and debugging purposes.

### 4.4. Data Loggers

**Figure 10:**
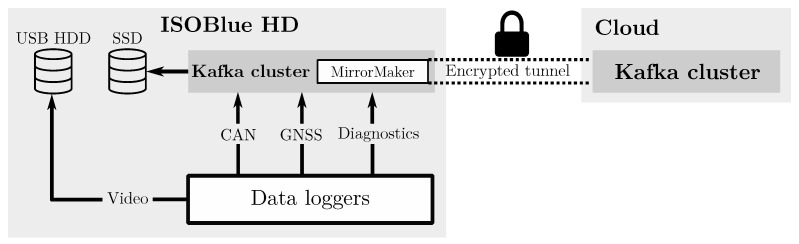
The Kafka cluster discussed in Section 4.3 was utilized for logging CAN, GNSS, and diagnostic data onto the SSD. The cluster communicated with a remote Kafka cluster via an encrypted tunnel for mirroring diagnostic information. Meanwhile, the RTSP video stream was saved directly to the USB HDD.

Different data loggers were developed to record four types of data, as shown in Figure 10. For logging CAN, GNSS, and diagnostic data, three custom Kafka producers were implemented: *kafka-can-log*, *kafka-gps-log*, and *heartbeat*. Two of these producers (*kafka-can-log* and *heartbeat*) were written in C and *kafka-gps-log* was written in Python. As a result, a mixture of middlewares in Table 6 were installed to provide runtime libraries to these loggers.

**Figure 11:**
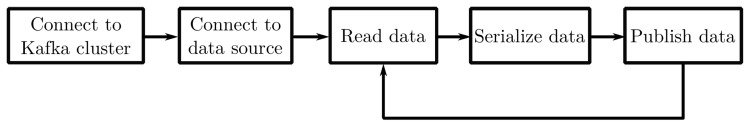
The Kafka data loggers share a similar workflow, yet differ in data sources. Each logger initializes a loop that continuously read, serialize, and publish data for data collection.

These applications share a common workflow, as illustrated in Figure 11. Each application starts by connecting to the Kafka cluster. It then attempts to connect to a data source and subsequently enters a loop for reading, serializing, and eventually publishing data to the Kafka cluster via Kafka client APIs. The differences stem from the data sources. Specifically, *kafka-can-log* creates a *SocketCAN* network socket for listening on a CAN bus; *kafka-gps-log* connects to *gpsd* via *gps3* for fetching GPS data. Meanwhile, *heartbeat* directly queries Internet connection status and cellular strength once per second using a system command. Once the data from different loggers are published to the Kafka cluster, they are stored in the SSD.

Meanwhile, three *systemd* services were written to automatically record the RTSP streams via *ffmpeg* [64]. Each service is responsible for connecting to the dedicated RTSP stream using an IP address and saving the stream to the USB HDD in 10-min blocks as audio video interleave (AVI) files. Each video file is named using the epoch timestamp when the recording starts. Moreover, it is worth noting that *ffmpeg* records raw video streams with no resizing or resampling. Hence, each resultant AVI file is able to retain the original video quality as described in Section 3.1.

## 5. Wheat Harvest Experiment

**Figure 12:**
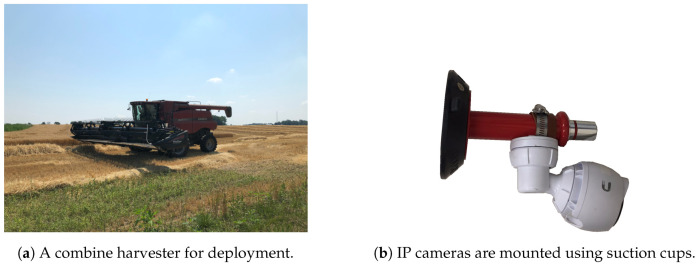
*ISOBlue HD* was deployed into a combine harvester for collecting a wheat harvest dataset. IP cameras were fastened onto heavy-duty suction cups for mounting on different surfaces.

*ISOBlue HD* was deployed in CASE IH Axial- Flow^®^ [65] 6088 combine harvester (Figure 12a) during wheat harvest in July 2019. The harvest took place in Rochester, Indiana, USA. The device was connected to the ISOBUS diagnostic port and placed in a nonintrusive location in the machine cab. The IP cameras were mounted in three in-cab positions: two on the front windshield and one on the back window behind the operator seat. The goal of the front cameras was to capture video on the header status, while the back camera was to record operator actions on a joystick and control panel buttons. The cameras were secured onto glass surfaces using heavy-duty suction cups, as shown in Figure 12b.

**Figure 13:**
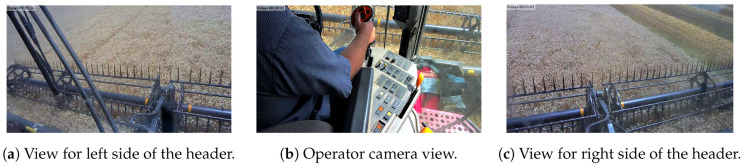
The tri-camera configuration captured header statuses and operator actions.

The device was retrieved at the end of the harvest for data offloading. The offloaded data contained a total of over 230 thousand GPS points, 10 million video frames, and 69 million CAN frames, which added up to 437 GB of data covering approximately 84 h of harvest time. Figure 13a–c illustrate sample frames captured from the three cameras. The rest of this section attempts to examine an hour-long context-rich data from 15 July 2019. It is noteworthy to point out the purpose of the following subsections is to highlight exploratory findings concerning the harvest and more importantly, present an extendable data processing template that integrates labeling, mining, and merging data sources within a context-rich dataset, as opposed to giving conclusive solutions or results on operational logistics and decision-making.

### 5.1. Contextual Label Generation

The video data were labeled for context according to two sets of contextual labels defined in Table 7. The first set encapsulates the header position of the combine harvester from the front camera views. On the other hand, the second set focuses on frequent joystick and control panel buttons that can be observed from the operator view.

**Figure 14:**
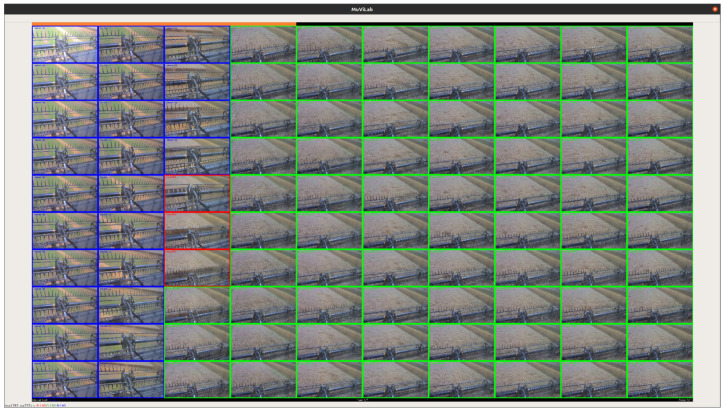
MuViLab, an open-source tool for simultaneously labeling multiple frames of a video, was utilized for labeling videos with contextual labels. In this screenshot of the tool interface, blue, red, green boxes each stand for “header up”, “transition”, and “header down” labels.

Each video was labeled by an individual with domain knowledge. This was achieved via an open-source tool called MuViLab [66]. The tool uses a set of labels and a video data file as inputs and automatically splits the video data into short clips. These clips are then played in a loop with a matrix-like format in an application interface demonstrated in Figure 14. The individual could then iteratively label each clip until the clips run out. After this process, the tool generates a file that contains a sequence of clip-label mappings. This file was further processed to output a file that associates a sequence of labels to corresponding epoch timestamps. This process was repeated for video files from three cameras. As a result, three time-series label files were generated.

### 5.2. Preliminary Contextual Knowledge

**Figure 15:**
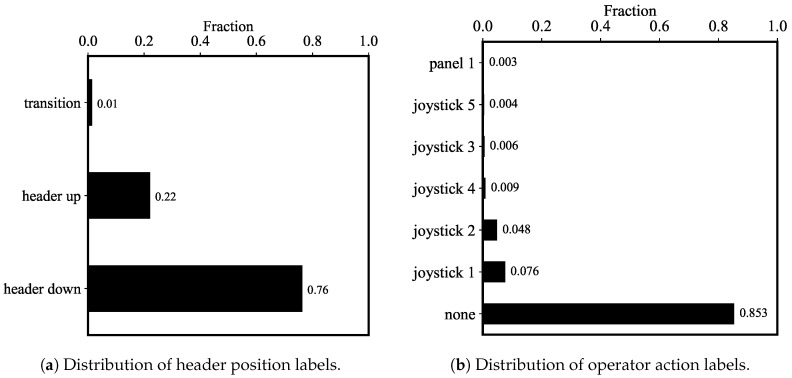
Fractions of labels give an idea of overall operations of the combine harvester. From (**a**), “header down” is the logical status for header position as the combine needs to lower the header to harvest. For (**b**), “joystick 1” and “joystick 2” has the second and third biggest fractions due to operator’s consistent adjustment of header reel height and header height when cutting wheat.

Preliminary contextual knowledge can be extracted from the time-series label files. Plots in Figure 15 visualize the fractions of the number of occurrences for header positions and operator actions. From Figure 15a, “header down” occurs more frequently than “header up” and “transition”, as the combine header usually stays down during harvest. Moreover, apart from the “none” label in Figure 15b, “joystick 1” (header height and tilt) and “joystick 2” (reel height) are the two most likely labels. This is likely due to the fact that the operator actively adjusts reel and header height throughout the harvesting process according to the crop status and the terrain.

**Figure 16:**
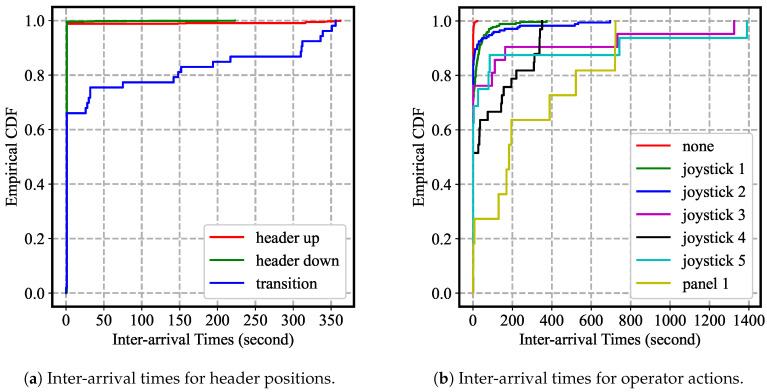
The empirical CDFs were estimated for the inter-arrival times for both sets of labels. Domain knowledge can be applied to explain the maximum of the inter-arrival times and the smoothness of the CDFs.

Besides, plots in Figure 16 reveal more information by examining the cumulative distribution functions (CDFs) of the inter-arrival times of labels. Figure 16a shows that both “header up” and “header down” labels have consistent yet short inter-arrival times. However, “header up” has a maximum of inter-arrival times of over 350 s while “header down” has a maximum of about 225 s. This difference in maximum is likely due to the difference in activities associated with these two labels. When the header is up, the combine is opted to transition to the next pass, unload crop, or park. On the other hand, when the header is down, the combine is normally limited to harvest crops. Hence, the additional flexibilities in activity with the “header up” label have increased its maximum inter-arrival times.

Furthermore, the smoothness of the CDF curves in Figure 16b suggests the discrepancies in the frequencies of operator actions on various joystick and control panel buttons. The relative smoothness of “joystick 1” and “joystick 2” CDFs suggests that these two actions are more frequent than the other actions. This not only corresponds to the relative high fraction of these two labels in Figure 15b, but also aligns with the heuristic experience that an operator consistently makes subtle adjustment on both header reel and header height according to the height of wheat during harvest.

### 5.3. GPS Track with Contexts

**Figure 17:**
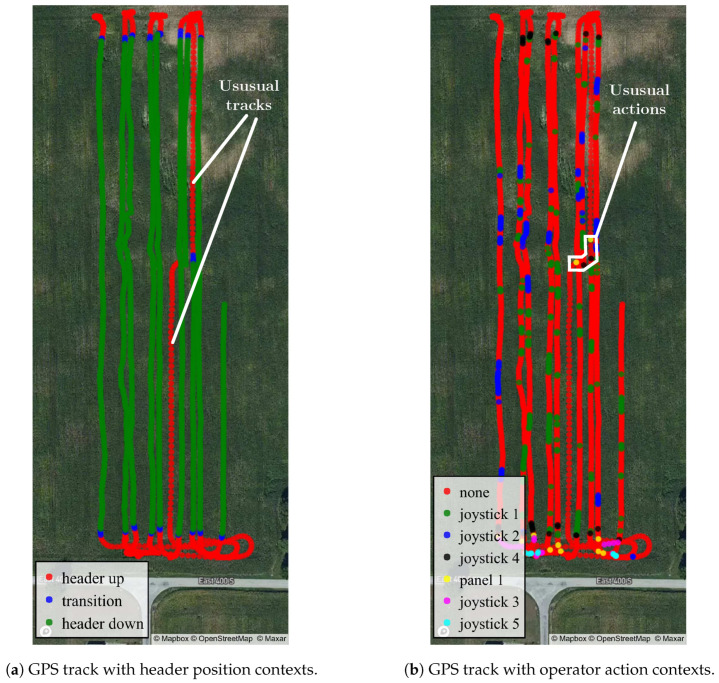
GPS track data, paired with contextual labels, can reveal information that cannot be easily obtained from GPS track only. For example, header position contexts provide a clear separation between harvest and non-harvest area in (**a**).

The combine’s GPS track visualizes where the machine has been during harvest. Analyzing the track alone reveals limited information on the navigation choice done by the operator. On the other hand, by incorporating contextual labels, the reasons behind certain choices could be inferred. For example, as shown in Figure 17, the one-hour GPS track comprising approximately nine passes for harvesting was merged with contextual labels based on timestamps. GPS coordinates in Figure 17a are highlighted using the header position labels. The “header down” coordinates indicate where the wheat is cut. Moreover, the “transition” coordinates provide a clear cut-off where the combine harvester stops harvesting. In addition, the “header up” coordinates suggest maneuvers such as turning, reversing, etc., in headlands. Apart from these obvious observations, by looking at Figure 17b jointly, the two unusual passes map to a sequence of unusual operator actions (header up/down and reel on/off) in the middle of the field, which could potentially be caused by a full grain bin or machine anomaly; an inference that could not be made without the contextual labels.

Figure 17b also highlights the relations between operator actions and geospatial coordinates. For instance, “joystick 1” (header height and tilt) and “joystick 2” (header reel height) occur frequently within each pass. Moreover, “joystick 4” (header reset/resume) only occurs during headland transitions. Actions “joystick 3” (unload auger extension) and “joystick 5” (auger on/off) are only triggered in headlands where a tractor-hauled grain cart is likely to park and wait for crop transfer. Moreover, the frequent usage of header height and reel height adjustments indicate the heavy involvement of human inputs during the harvesting process, which could be sources of error and fatigue.

### 5.4. Extracted CAN Signal with Contexts

CAN data typically contain straightforward information about machine and operation status. However, the proprietary nature of CAN data renders most research effort exclusively to few OEM engineers and ones who have access to the decoding information. Nevertheless, by leveraging labels generated in Section 5.1, the following example offers a possibility to understand unknown CAN data, from both a contextual and non-proprietary perspective; or even reverse-engineer the unknowns for security concerns.

**Figure 18:**
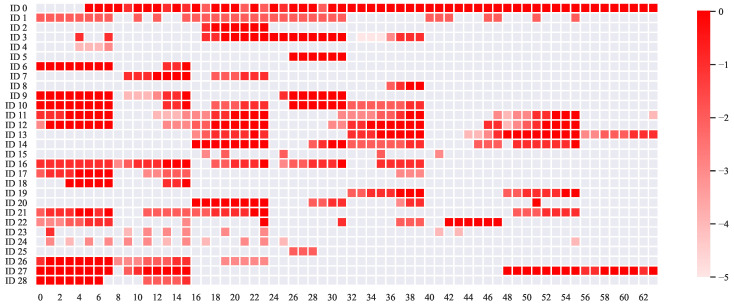
Bit-flip magnitudes for 29 unique CAN IDs are visualized. A larger magnitude value represents a more frequent change for a particular bit.

In this example, the CAN data were first examined by their payloads before merging with contextual labels. A logged CAN frame consists of a timestamp, a CAN ID, and a 64-bit payload. There are 29 unique CAN IDs in the dataset. These CAN IDs are usually associated with decoding schemes for extracting sensory data from the payloads—a critical intermediate step in studying machine status. However, CAN IDs and their underlying decoding schemes for their payloads are mostly proprietary. Various recent works [67,68,69,70,71,72] provide methods that compute the bit-flip rate and the bit-flip magnitude of each bit in the payload for estimation of payload boundaries, without the reliance of proprietary information. Once boundaries had been identified, sensor data could be subsequently extracted. Following previous works, the bit-flip rate *B* for a particular bit of the 64-bit payload was obtained by dividing the total number of bit-flips over the total number of payloads for a CAN ID. The bit-flip magnitude for a particular bit is defined as $\left\lceil \log_{10}\left(B\right)\right\rceil$. Using these definitions, bit-flip rates were first computed for all 29 CAN IDs. Bit-flip magnitudes were then evaluated and visualized in Figure 18.

**Figure 19:**
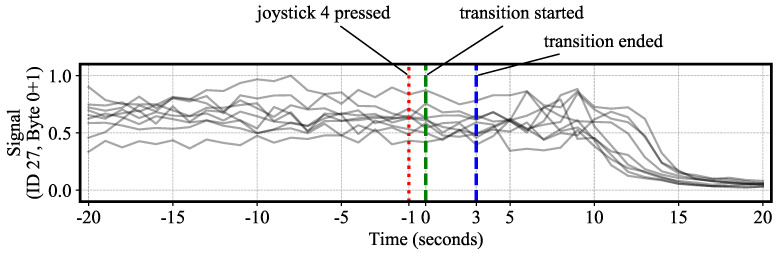
Extracted signal traces from CAN ID 27 are visualized with both selected header position and operation action labels. By incorporating context, the meaning of the signal traces can be interpreted as flow or yield sensor readings.

Physical signals can be extracted by visually inspecting and locating the payload boundaries from Figure 18. The extracted signals were further merged with contextual labels to uncover signal semantics with no prior knowledge about what the signal means. For instance, Figure 19 plots a series of CAN ID 27 signal traces (by concatenating byte 0 and 1) 20 s before and after the header down-to-up transition occurs. The figure also plots the average time instances when the operator presses “joystick 4” and when the header finishes transitioning to a down position. It can be observed that there is consistently approximately a one-second delay in between “joystick 4” presses and the start of each transition. In addition, the time needed for the header up to transition to down is on average 3 s. Moreover, after the header is fully in a down position, the signal traces gradually fall to 0 after a roughly 12-s delay. This phenomenon suggests that information encapsulated in byte 0 and 1 of CAN ID 27 could originate from a yield or flow sensor, since the operator typically keeps the thresher on at the end of each pass for an extended time to thresh the last bit of crop. Although this suggestion is heuristic and speculative, it could not have been generated without combining contextual labels with CAN data.

## 6. Conclusions

*ISOBlue HD* successfully collected CAN, GPS, and video data from a combine harvester during a 2019 wheat harvest. The video data captured header status and operator actions. The dataset was processed to generate preliminary context-rich results. The video data were first labeled with header position and operation action labels to generate time-series contextual label files. These contextual labels were analyzed to generate preliminary knowledge of the distributions and frequencies of different labels. Moreover, the contextual labels were merged with CAN and GPS data based on timestamps. Both CAN and GPS data reveal unique perspectives with contextual labels. GPS coordinates highlighted with header position labels provided clear distinctions between harvest vs. non-harvest areas. On the other hand, the extracted CAN signal was paired with both header position and operator action labels to infer its meaning, with no prior knowledge of the signal.

Despite the success in deployment and data collection, both the platform and the post-processing pipeline have room for improvement in future works. First, the enclosure platform was bulky and inconvenient to move around in the cab. Its size could be reduced and more efforts are needed to make a sealed enclosure for in-cab or even cabless environment. Meanwhile, the platform experienced inconsistent Internet access, which caused delayed data streaming and failed remote login attempts on multiple occasions. Although sporadic network access is a common problem in rural areas [73], an intelligent data streaming policy needs to be implemented to batch data during sub-optimal network conditions and send data opportunistically whenever the network becomes available. Moreover, although the Kafka cluster performed well for storing data, it was cumbersome to fetch data from the cluster during post-processing, as the logs could not be queried like a traditional database. Hence, it would be reasonable to replace Kafka with a more compact database that provides both indexed data logging as well as querying capabilities. In addition, for scaling up the experiment, multiple *ISOBlue HDs* could be deployed in different machines for setting up a distributed context-rich sensing network that enables the real-time inference of operational contexts as well as potential context sharing. This potentially needs more minable data from other operations (tilling, spraying, etc.) from a fleet of different machines. Lastly, to realize context-sharing, an efficient data transmission scheme that involves proper compression, error control, etc. is essential in ensuring the reliable sharing of inferred contexts among machines in rural areas. Limited network connectivity in these areas requires, for instance, eliminations of unwanted redundancy of CAN payload bits to save transmission bandwidth, which could be an extension of the work discussed in Section 5.4.

In terms of post-processing, the manual data processing steps in Section 5 could be enhanced in a couple of aspects; much of them could better be addressed with specific applications. For instance, one application of improving machine function comes directly from the data discussed in Section 5.3; the extremely frequent adjustments of joysticks 1 and 2 (header height and tilt, reel height) are a major source of fatigue. Artificial intelligence developments to capitalize on video processing and other available layers (perhaps aerial imagery, topography, knowledge of the neighboring pass) to automate these adjustments could improve safety along with operation effectiveness. This requires the automatic labeling and processing of video data from different camera views using computer vision algorithms, as highlighted in works like [74,75] for studying the relations among hand/finger movements, header positions/motions, and overall harvesting efficiency. This knowledge could be further combined with geospatial and CAN data for pushing machine performance “to the limit”, in terms of optimized path planning and engine capacity without sacrificing threshing and cleaning quality. All of these insights rely on a comprehensive data pipeline—the focus of future works, which encapsulates research topics like GNSS track clustering, signal extraction/interpretation from CAN data, and sensor signal classification [76].

## Figures and Tables

**Table 1:** A list of selected custom hardware platforms for agricultural machinery data collection.

Name	Collected Data	Reference
CyCAN	CAN	Darr [28]
ISOBlue	CAN	Layton et al. [29]
CANdroid	CAN	Wang et al. [30]
ISOBlue 2.0	CAN, GNSS	Wang et al. [31]
FieldSafe	GNSS, video, IMU, ranging	Kragh et al. [32]
PolyCAN	CAN	Fite et al. [33]
Cropinfra	CAN, GNSS	Backman et al. [34]

**Table 2:** Highlights of single board computer specifications.

Feature	Description
Processor	ARM^®^ Cortex-A9, 800 MHz, quad-core
RAM	2 GB DDR3
Flash	4 GB eMMC flash
Interfaces	mSATA, miniPCIe, Ethernet, CAN, USB, GPIO
Input power	7 to 27 V

**Table 3:** Specifications of data sources illustrated in Figure 4.

Component	Quantity	Model	Description	Acquisition Rate (Nominal)
CAN transceiver	2	Analog Device ADM3053 [38]	Converts CAN bus signals to bitstreams.	700 frames/second
USB GPS module	1	Navisys GR-701W [39]	Provides positioning data with 2.5 m accuracy.	1 message/second
IP camera	3	Ubiquiti Networks UVC-G3-BULLET [40]	Provides full HD (1080p) video streams.	30 frames/second

**Table 4:** A list of system management applications.

Application	Description
udev [48]	System device manager
systemd [50]	System service daemon
openssh [51]	Networking tools using Secure Shell (SSH)
dnsmasq [52]	Multi-purpose networking tool
chronyd [53]	Clock synchronization daemon
gpsd [49]	GPS service daemon

**Table 5:** Data stored in different Kafka topics.

Topic	Description
imp	Implement bus data
tra	Tractor bus data
gps	GPS data
remote	GPS and diagnostic data

**Table 6:** Preinstalled middlewares for the Kafka data loggers.

Name	Description
librdkafka [59]	Apache Kafka client C API
kafka-python [60]	Apache Kafka client Python API
avro-c [61]	Apache Avro serialization C library
avro-python [62]	Apache Avro serialization Python library
gps3 [63]	Client Python library for gpsd

**Table 7:** Header position and operator action contextual labels.

Label	Description
*Header position*	
Header up	Header is at up position.
Transition	Header transition (up to down or vice versa).
Header down	Header is at down position.
*Operator actions*	
None	No operator action observed.
Joystick 1	Header height and tilt.
Joystick 2	Reel height adjustment.
Joystick 3	Unload auger swing out/in.
Joystick 4	Resume header preset or raise header.
Joystick 5	Unload auger on/off.
Panel 1	Header rotation on/off.

## Appendix A

**Table A1:** Bill of Materials for hardware components utilized in *ISOBlue HD*.

Component	Model	Quantity	Unit Cost (USD)
*Single board computer*	
Ixora carrier board	Toradex 01331000	1	$99
Apalis computer module	Toradex 00281101	1	$139
*Sensors*	
IP cameras	Ubiquiti Networks UVC-G3-BULLET	3	$149
USB GPS module	Navisys GR-701W	1	$49
CAN transceiver (on Ixora)	Analog Device ADM3053BRWZ	2	$0
*Data storage*	
mSATA solid state drive	Samsung PM851	1	$299
USB hard drive	Western Digital WDBYFT0040BBK	1	$81
*Cellular connectivity*	
miniPCIe 4G/LTE module	Telit LE910-NAG	1	$95
Antennas	Taoglas FXUB65-07-0180C	2	$10
*Power related*	
Relay PCB	Custom built	1	$10
RTC battery	CR 1225	1	$1
Network switch	Veracity VCS-8P2-MOB	1	$248
*Enclosure*	
IP 68 Enclosure	Integra H12106SCF	1	$193
Plexiglass	Custom built	1	$10
Ethernet coupler	Tripp Lite N206-BC01-IND	3	$14
USB coupler	USBFirewire RR-111200-30	1	$15
ISOBUS coupler	Deutsch HD10-9-1939PE-B022	1	$10
Cablings	Various vendors	N/A	$5
